# THE ASSOCIATION BETWEEN NUMBER OF FRIENDS AND LONELINESS FOR OLDER MEN AND WOMEN USING THE HEALTH AND RETIREMENT STUDY

**DOI:** 10.1093/geroni/igad104.1586

**Published:** 2023-12-21

**Authors:** Emily Lim, Jeffrey Burr

**Affiliations:** University of Massachusetts Boston, Boston, Massachusetts, United States; University of Massachusetts Boston, Boston, Massachusetts, United States

## Abstract

Loneliness is a significant health risk. Older adults are susceptible to loneliness and friendship is a source of support, potentially alleviating loneliness in later life. Few studies examined the association between friendship and loneliness among older adults and whether gender moderates this association. We examined the association between friendship and loneliness for older men and women longitudinally with data from the Health and Retirement Study (2006, 2010, 2014, 2018). Our sample included 7,257 community-dwelling adults aged 50 and above (M=68.05, SD=10.38). Using multilevel linear modeling, we examined the effects of number of friends, including quadratic and cubic terms for number of friends. After controlling for covariates, our results showed having more friends is negatively associated with loneliness (β=-0.21, p<.001) and for the quadratic friends term (β=0.01, p<.001), and the cubic friends term (β=-0.00, p<.01) on loneliness. While the number of friends is also negatively associated for both men (β=-0.15, p<.001) and women (β=-0.30, p<.001), there were significant effects of the quadratic (β=0.04, p<.001) and cubic friends terms on loneliness for women (β=-0.00, p<.001). For men, only the quadratic friends term was significant (β=0.02, p<.001) but not the cubic friends term. These results show there is a non-linear relationship between number of friends and loneliness, with a possible threshold effect for men and a more complex relationship for women. Our findings have implications for friendship in later life, indicating that social interventions may need to be tailored differently for older men and women to promote friendships and potentially reduce loneliness.

